# Novel Cycloheximide Derivatives Targeting the Moonlighting Protein Mip Exhibit Specific Antimicrobial Activity Against *Legionella pneumophila*

**DOI:** 10.3389/fbioe.2015.00041

**Published:** 2015-03-27

**Authors:** Janine Rasch, Martin Theuerkorn, Can Ünal, Natascha Heinsohn, Stefan Tran, Gunter Fischer, Matthias Weiwad, Michael Steinert

**Affiliations:** ^1^Institut für Mikrobiologie, Technische Universität Braunschweig, Braunschweig, Germany; ^2^Max Planck Institute of Biophysical Chemistry Göttingen BO Halle, Halle, Germany; ^3^Türk-Alman Üniversitesi, Fen Fakültesi, Istanbul, Turkey; ^4^Institut für Biochemie und Biotechnologie, Universität Halle-Wittenberg, Halle-Wittenberg, Germany; ^5^Helmholtz Centre for Infection Research, Braunschweig, Germany

**Keywords:** moonlighting, PPIase, cycloheximide, adamantane, inhibitor

## Abstract

Macrophage infectivity potentiator (Mip) and Mip-like proteins are virulence factors in a wide range of pathogens including *Legionella pneumophila*. These proteins belong to the FK506 binding protein (FKBP) family of peptidyl-prolyl-*cis/trans*-isomerases (PPIases). In *L. pneumophila*, the PPIase activity of Mip is required for invasion of macrophages, transmigration through an *in vitro* lung–epithelial barrier, and full virulence in the guinea pig infection model. Additionally, Mip is a moonlighting protein that binds to collagen IV in the extracellular matrix. Here, we describe the development and synthesis of cycloheximide derivatives with adamantyl moieties as novel FKBP ligands, and analyze their effect on the viability of *L. pneumophila* and other bacteria. All compounds efficiently inhibited PPIase activity of the prototypic human FKBP12 as well as Mip with IC_50_-values as low as 180 nM and 1.7 μM, respectively. Five of these derivatives inhibited the growth of *L. pneumophila* at concentrations of 30–40 μM, but exhibited no effect on other tested bacterial species indicating a specific spectrum of antibacterial activity. The derivatives carrying a 3,5-dimethyladamantan-1-[yl]acetamide substitution (MT_30.32), and a 3-ethyladamantan-1-[yl]acetamide substitution (MT_30.51) had the strongest effects in PPIase- and liquid growth assays. MT_30.32 and MT_30.51 were also inhibitory in macrophage infection studies without being cytotoxic. Accordingly, by applying a combinatorial approach, we were able to generate novel, hybrid inhibitors consisting of cycloheximide and adamantane, two known FKBP inhibitors that interact with different parts of the PPIase domain, respectively. Interestingly, despite the proven Mip-inhibitory activity, the viability of a Mip-deficient strain was affected to the same degree as its wild type. Hence, we also propose that cycloheximide derivatives with adamantyl moieties are potent PPIase inhibitors with multiple targets in *L. pneumophila*.

## Introduction

The Gram-negative bacterium *Legionella pneumophila* is the causative agent of Legionnaires’ disease, a severe community acquired, and atypical pneumonia with mortality rates up to 5–30% despite appropriate antibiotic treatment (Fields et al., 2002). The virulence factor macrophage infectivity potentiator (Mip) of *L. pneumophila* is important for the establishment of the intracellular infection (Cianciotto et al., 1989; Cianciotto and Fields, 1992). It is a homodimeric lipoprotein that is localized on the surface of the bacteria (Helbig et al., 2001; Köhler et al., 2003). The dimerization occurs via its N-terminus that is followed by a long and flexible α-helix, and a C-terminal peptidyl-prolyl-*cis/trans*-isomerase (PPIase) domain that catalyzes the *cis/trans* interconversion of Xaa-Pro peptide bonds (Lang et al., 1987; Fischer et al., 1989; Riboldi-Tunnicliffe et al., 2001). Mip belongs to the family of FK506 binding proteins (FKBP), and its PPIase domain is structurally highly similar to human FKBP12. Accordingly, both proteins interact with the natural inhibitors FK506 and rapamycin in a similar way (Ceymann et al., 2008).

Mip was shown to be the first virulence associated PPIase (Fischer et al., 1992). Mip-negative mutants are delayed in the onset of the intracellular replication cycle in several host cell types like macrophage cell lines, alveolar macrophages, blood monocytes, lung epithelial cells, and protozoan hosts (Cianciotto et al., 1989, 1990, 1995; Cianciotto and Fields, 1992; Wintermeyer et al., 1995). Beyond monocellular hosts, Mip contributes to bacterial replication as well as dissemination within the lung tissue, and subsequent spread to the spleen in guinea pigs (Engleberg et al., 1989; Cianciotto et al., 1990; Cianciotto and Fields, 1992). This process could be simulated, and the contribution of Mip confirmed in *in vitro* transmigration assays. Furthermore, degradation assays with ^35^S-labeled ECM proteins support the hypothesis of a concerted action of Mip and proteolytic enzymes of either host or bacterial origin (Wagner et al., 2007).

Mip exerts part of its virulence associated functions in the lung by moonlighting as it can interact with collagen IV (Wagner et al., 2007). This interaction is mediated by the PPIase domain, which binds to a distinct 13-mer sequence within the non-collagenous domain (NC1) of the α1 isomer of collagen IV, and promotes transmigration of the bacteria across barriers constituted by collagen containing extracellular matrices (ECM) or lung epithelial cell lines (Wagner et al., 2007; Ünal et al., 2011). Hence, attenuation of Mip-deficient mutant bacteria in the guinea pig infection model is partly influenced by the moonlighting properties of Mip that facilitates adhesion to the ECM followed by efficient colonization and destruction of lung tissue.

Currently, moonlighting proteins that contribute to bacterial infections arise as interesting new therapeutic targets (Henderson and Martin, 2014). Since homologs of Mip are also present in other intracellularly replicating and persisting pathogens like *Trypanosoma cruzi*, *Coxiella burnetii*, *Neisseria gonorrhoeae*, and several members of the genus *Chlamydia*, it is suggested that moonlighting Mip and Mip-related PPIases are potential targets for novel antimicrobials (Lundemose et al., 1993; Mo et al., 1995; Moro et al., 1995; Rockey et al., 1996; Leuzzi et al., 2005). The characterized inhibitors rapamycin and FK506 are not suitable antibiotics, because they also interact with human FKBPs. Complexes of human FKBP12 and FK506 dock to the protein phosphatase calcineurin, thereby blocking T cell proliferation via the NFAT pathway (Liu et al., 1992). Moreover, the FKBP12-rapamycin complex inhibits an essential Ser/Thr protein kinase called mammalian target of rapamycin (mTOR), thereby influencing IL-2 receptor signal transduction (Boytim et al., 2000). As a result, both drugs have immunosuppressive properties that find clinical use mainly during organ transplantations (Gaali et al., 2011).

**Figure 1:**
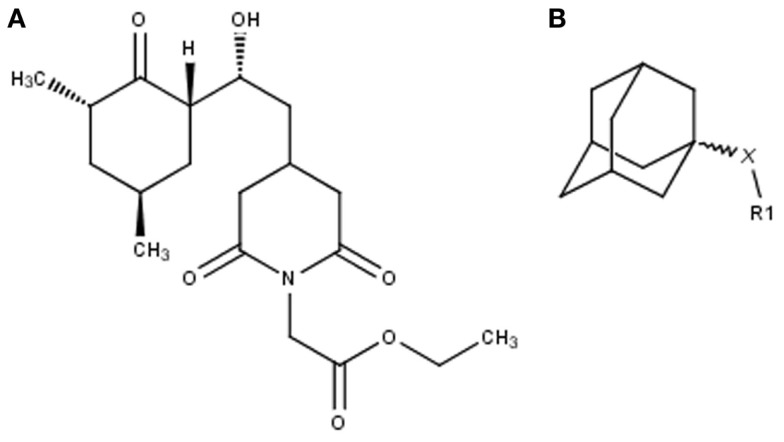
**Non-macrolide FKBP-inihibitors**. Shown are the two main building blocks of the novel FKBP inhibitors presented in this study. **(A)** Cycloheximide *N*-ethylethanoate is a non-toxic derivative of the natural compound cycloheximide, an inhibitor of eukaryotic translation. **(B)** Adamantane was identified by structure based computational modeling as an appropriate scaffold that can replace the pipecolinic acid moiety in FK506, which interacts with the PPIase active cleft of FKBPs. Adamantane and substituents of it were fused to *N*-(carboxymethyl)cycloheximide in order to produce novel cycloheximide derivatives (see also Table 1).

The search for non-immunosuppressive FKBP inhibitors has resulted in the discovery, or *de novo* design of several non-macrolide structures (Ünal and Steinert, 2014). Among those is the natural compound cycloheximide of *Streptomyces griseus* that was identified by screening a compound library. While cycloheximide is an efficient inhibitor of eukaryotic protein synthesis, its imide-substituted derivatives, such as cycloheximide *N*-ethylethanoate (Figure 1A) or *N*-(*N*′,*N*′-dimethylcarboxamido-methyl)-cycloheximide (DM-CHX), are devoid of this side effect, and potently inhibit FKBPs (Christner et al., 1999; Edlich et al., 2006; Norville et al., 2011). In contrast, adamantane and its derivatives (Figure 1B) were identified and produced in a custom tailored process by computational predictions based on the structure of the FKBP12-FK506 complex (Babine et al., 1995). Interestingly, these two substance classes seem to interact with FKBPs in different ways. While cycloheximide *N*-ethylethanoate primarily binds to the vicinity of the enzymatic cleft, adamantane and its derivatives can replace the pipecolinic acid moiety of FK506 making close contact to several conserved amino acids in the PPIase pocket (Babine et al., 1995; Norville et al., 2011). The aims of the present study were the synthesis of novel Mip ligands by the combination of both substance classes and the evaluation of their PPIase inhibitory action as well as their spectrum of antibacterial activity and potential as suppressors of *Legionella*-infection.

## Materials and Methods

### Materials for chemical synthesis

All chemicals, unless otherwise noted, were purchased from Sigma-Aldrich and were of reagent grade. Trifluoroacetic acid (TFA) was obtained from Merck. FK506 and cyclosporine A (CsA) were obtained from LC Laboratories. Acetonitrile (ACN) HPLC gradient grade was purchased from Fisher Scientific.

### Instrumentation

The preparative HPLC Sykam contained two S1021 pumps, a controller S2001, an Abimed 118 UV/VIS detector and reverse phase columns Phenomenex Gemini-NX 250 × 21.2 C18, and a Phenomenex Jupiter Proteo 90A AX 250 × 21.2. Solvent A: H_2_O. Solvent B: ACN. The purity of the final products was determined using a Dionex analytical HPLC unit coupled with a photodiode array detector and a Vydac reverse phase column (C18 5 μm, 4.6 mm × 250 mm column) coupled with a Surveyor MSQ (Thermo Finnigan) mass spectrometer. The same solvent system used for preparative HPLC was used for analytical HPLC. Demineralized water was additionally purified with a Millipore water purification system.

### Chemical synthesis

#### *N*-(carboxymethyl)cycloheximide

To a solution of cycloheximide (500 mg; 1.78 mmol) in 15 ml of DMF was added tert-butylbromo acetate (369 μl; 2.5 mmol) and anhydrous K_2_CO_3_ (442 mg; 3.2 mmol), and the solution was stirred overnight at room temperature. After evaporation of the solvent, the resulting residue was dissolved in ethyl acetate and filtered following evaporation of ethyl acetate.

The entire crude product *N*-[methyl-(tert-butyloxycarbonyl)] cycloheximide was dissolved in 20 ml of CH_2_Cl_2_, and 4 ml of a 2.2M zinc (II)-chloride diethyl etherate solution (freshly prepared) was added and stirred at room temperature for 4 h. The mixture was transferred into a separatory funnel and washed with a saturated aqueous ETDA solution (2 ml × 10 ml). The layers were separated and the organic phase was washed an additional time with 10 ml of a saturated aqueous NaCl solution. The organic layer was separated, dried over Na_2_SO_4_, and the solvent was evaporated. The crude product was used without further purification. The crude *N*-(carboxymethyl)cycloheximide was achieved with a yield of 479 mg (79% relating to applied amount of cycloheximide). [M + H] + calc. = 340.2, [M + H] + found = 340.4.

#### MT_30.32

*N*-(carboxymethyl)cycloheximide (10.0 mg; 29.5 μmol), PyBOP (16.0 mg; 29.5 μmol), and *N*-ethyldiisopropylamine (15.0 μl; 74.5 μmol) were dissolved in 4 ml DMF and pre-incubated for 5 min. 3,5-dimethyl-1-adamantamine hydrochloride (12.0 mg; 59.0 μmol) was added and the mixture was stirred for 1 h at room temperature. The solvent was evaporated and the crude product was purified via preparative HPLC using a RP C18 column (Phenomenex Gemini-NX, 250 × 21.2) with a linear gradient of 30–80% ACN in 40 min and a flow rate of 17.0 ml/min. The product was achieved with a yield of 6.7 mg (45.5%). [M + H] + calc. = 501.3, [M + H] + found = 501.5.

#### MT_30.51

*N*-(carboxymethyl)cycloheximide (10.0 mg; 29.5 μmol), PyBOP (16.0 mg; 29.5 μmol), and *N*-ethyldiisopropylamine (15.0 μl; 74.5 μmol) were dissolved in 4 ml DMF and pre-incubated for 5 min. (3-ethyl-1-adamantyl)amine hydrochloride (12.8 mg; 59.0 μmol) was added and the mixture was stirred for 1 h at room temperature. The solvent was evaporated and the crude product was purified via preparative HPLC using a RP C18 column (Phenomenex Gemini-NX, 250 × 21.2) with a linear gradient of 20–60% ACN in 30 min and a flow rate of 17.0 ml/min. The product was achieved with a yield of 8.6 mg (58.5%). [M + H] + calc. = 501.3, [M + H] + found = 501.4.

#### MT_30.38

*N*-(carboxymethyl)cycloheximide (10.0 mg; 29.5 μmol), PyBOP (16.0 mg; 29.5 μmol), and *N*-ethyldiisopropylamine (10.4 μl; 59.0 μmol) were dissolved in 4 ml DMF and pre-incubated for 5 min. 3-Amino-1-adamantol (9.8 mg; 59.0 μmol) was added and the mixture was stirred for 1 h at room temperature. The solvent was evaporated and the crude product was purified via preparative HPLC using a RP C18 column (Phenomenex Gemini-NX, 250 × 21.2) with a linear gradient of 30–80% ACN in 40 min and a flow rate of 17.0 ml/min. The product was achieved with a yield of 7.2 mg (50.0%). [M + H] + calc. = 489.3, [M + H] + found = 489.3.

#### MT_30.79

*N*-(carboxymethyl)cycloheximide (10.0 mg; 29.5 μmol), PyBOP (16.0 mg; 29.5 μmol), and *N*-ethyldiisopropylamine (10.4 μl; 59.0 μmol) were dissolved in 4 ml DMF and pre-incubated for 5 min. 3-amino-5,7-dimethyl-adamant-1-ol (11.5 mg; 59.0 μmol) was added and the mixture was stirred for 1 h at room temperature. The solvent was evaporated and the crude product was purified via preparative HPLC using a RP C18 column (Phenomenex Gemini-NX, 250 × 21.2) with a linear gradient of 30–80% ACN in 40 min and a flow rate of 17.0 ml/min. The product was achieved with a yield of 6.3 mg (41.4%). [M + H] + calc. = 517.4, [M + H] + found = 517.3.

#### MT_30.3

*N*-(carboxymethyl)cycloheximide (10.0 mg; 29.5 μmol), PyBOP (16.0 mg; 29.5 μmol), and *N*-ethyldiisopropylamine (10.4 μl; 59.0 μmol) were dissolved in 4 ml DMF and pre-incubated for 5 min. Tert-butylamine (6.2 μl; 59.0 μmol) was added and the mixture was stirred for 1 h at room temperature. The solvent was evaporated and the crude product was purified via preparative HPLC using a RP C18 column (Phenomenex Gemini-NX, 250 × 21.2) with a linear gradient of 30–80% ACN in 40 min and a flow rate of 17.0 ml/min. The product was achieved with a yield of 5.9 mg (50.8%). [M + H] + calc. = 395.2, [M + H] + found = 395.3.

#### MT_30.9

*N*-(carboxymethyl)cycloheximide (10.0 mg; 29.5 μmol), PyBOP (16.0 mg; 29.5 μmol), and N-ethyldiisopropylamine (10.4 μl; 59.0 μmol) were dissolved in 4 ml DMF and pre-incubated for 5 min. Tert-butylmethylamine (7.0 mg; 59.0 μmol) was added and the mixture was stirred for 1 h at room temperature. The solvent was evaporated and the crude product was purified via preparative HPLC using a RP C18 column (Phenomenex Gemini-NX, 250 × 21.2) with a linear gradient of 30–80% ACN in 40 min and a flow rate of 17.0 ml/min. The product was achieved with a yield of 9.2 mg (76.7%). [M + H] + calc. = 409.2, [M + H] + found = 409.4.

#### MT_30.8

*N*-(carboxymethyl)cycloheximide (10.0 mg; 29.5 μmol), PyBOP (16.0 mg; 29.5 μmol), and *N*-ethyldiisopropylamine (10.4 μl; 59.0 μmol) were dissolved in 4 ml DMF and pre-incubated for 5 min. 1-aminoadamantan (9.0 mg; 59.0 μmol) was added and the mixture was stirred for 1 h at room temperature. The solvent was evaporated and the crude product was purified via preparative HPLC using a RP C18 column (Phenomenex Gemini-NX, 250 × 21.2) with a linear gradient of 30–80% ACN in 40 min and a flow rate of 17.0 ml/min. The product was achieved with a yield of 8.7 mg (62.6%). [M + H] + calc. = 473.3, [M + H] + found = 473.5.

#### MT_30.92

(S)-2-methyl-CBS-oxazaborolidine (1.2 mg; 21.2 μmol) and a 1M solution of BH3 in THF (22.0 μml; 21.2 μmol) were dissolved in 5 ml of anhydrous THF and stirred for 15 min. This solution was added slowly within 30 min to a stirred solution of MT_30.8 (10.0 mg; 21.2 μmol) in 10 ml anhydrous THF at 0°C. The stirring is maintained for 40 min. Thereafter, 10 ml of methanol was added and the solution was evaporated. The crude product was purified via preparative HPLC using a RP C12 column (Phenomenex Gemini-NX, 250 × 21.2) with a linear gradient of 30–80% ACN in 40 min and a flow rate of 17.0 ml/min. The product was achieved with a yield of 5.8 mg (58.0%). [M + H] + calc. = 475.3, [M + H] + found = 475.5.

#### MT_30.93

(R)-2-methyl-CBS-oxazaborolidine (1.2 mg; 21.2 μmol) and a 1M solution of BH3 in THF (22.0 μml; 21.2 μmol) were dissolved in 5 ml of anhydrous THF and stirred for 15 min. This solution was added slowly within 30 min to a stirred solution of MT_30.8 (10.0 mg; 21.2 μmol) in 10 ml anhydrous THF at 0°C. The stirring is maintained for 40 min. Thereafter, 10 ml of methanol was added and the solution was evaporated. The crude product was purified via preparative HPLC using a RP C12 column (Phenomenex Jupiter Proteo 90A AX, 250 × 21.2) with a linear gradient of 30–80% ACN in 40 min and a flow rate of 17.0 ml/min. The product was achieved with a yield of 6.9 mg (69.1%). [M + H] + calc. = 475.3, [M + H] + found = 475.3.

### Protease coupled PPIase assay

The PPIase activity of Mip or FKBP12 was determined by using the peptide substrate Succinyl-Ala-Leu-Pro-Phe-4-nitroanilide in the protease-coupled PPIase assay as described previously (Fischer et al., 1992). Briefly, 30 nM Mip or 8 nM FKBP12 was preincubated with 40 μM peptide substrate in the presence or absence of various inhibitor concentrations for 4 min at 10°C in 35 mM HEPES/NaOH buffer (pH 7.8). Then, reaction was started by addition of 100 μg/mL of the isomer specific protease chymotrypsin, and the release of 4-nitroaniline was monitored by measuring the absorbance at 390 nm.

### Determining the minimal inhibitory concentration

The minimal inhibitory concentration (MIC) method was used to determine the minimal concentration of cycloheximide derivatives that is needed to inhibit the growth of *L. pneumophila* Corby*, Yersinia pseudotuberculosis, Escherichia coli* K12*, Staphylococcus aureus, Pseudomonas aeruginosa* PA01*, Salmonella enterica* subsp. *Enterica*, and *Klebsiella aerogenes*. All bacteria were cultured in LB (Luria-Bertani) medium except for *L. pneumophila*, which were grown in YEB medium (buffered yeast extract broth). All bacterial species were cultured overnight at 37°C and 200 rpm, and adjusted to 2 × 10^7^ cells/ml with LB or YEB, respectively. One hundred microliter aliquots of these bacterial suspensions were transferred to 96 well plates and were mixed with an additional 100 μl medium with and without several cycloheximide derivatives starting at concentrations between 10 and 100 μM concentrations with 10 μM increments. Bacteria were cultured at 37°C and 180 rpm for 2 days, and growth was measured at 600 nm in a microplate reader.

### *In vitro* activity of cycloheximide derivatives in infected macrophages

The human monocyte cell line THP-1 (ACC 16) was purchased from the German Collection of Microorganisms and Cell Cultures (The Leibniz Institute DSMZ), and maintained in RPMI supplemented with 10% fetal bovine serum and 2 mM l-glutamine at 37°C in a 5% CO_2_-atmosphere. For infection assays, the monocytes were adjusted to 5 × 10^5^ cells/ml in fresh medium supplemented with 100 nM phorbol-12-myristate-13-acetate (PMA, Sigma–Aldrich P8139). Two hundred microliters of this suspension were transferred into the wells of a 96-well cell culture plate, and the cells were differentiated for 2 days into macrophage-like cells.

For infection, *L. pneumophila* Corby was grown over night in YEB medium to early stationary phase. Bacteria were washed once with H_2_O_dd_ (3 min, 6000 × *g*, RT), and adjusted to 10^6^ cfu/ml in fresh RPMI (infection medium). The medium of differentiated THP-1 cells was replaced by 100 μl of infection medium (MOI 1). After 2 h, extracellular bacteria were removed by washing three times with pre-warmed PBS. Finally, the cells were covered with fresh medium containing cycloheximide derivatives at the indicated concentrations. Medium containing only 1% (v/v) ethanol or 1% (v/v) DMSO was used as control. Uptake of bacteria and bacterial replication were monitored by lysing the cells after 2 and 24 h after infection, respectively, by adding Triton X-100 at a final concentration of 0.1% (v/v), and plating out serial dilutions on BCYE agar plates.

### Cytotoxicity assay

In order to rule out cytotoxic side effects of the biologically active cycloheximide derivatives on eukaryotic cells, the viability of THP-1 cells was measured using the Alamar Blue reagent (Biozol, #BZL00726) according to manufacturer’s instructions. Briefly, THP-1 cells were prepared as described above. Instead of infecting the cells, they were incubated with 100 μM MT_30.32, or MT_30.51 for 20 h, at which time point Alamar Blue reagent was added. After another 4 h, cell viability was assessed by measuring fluorescence intensity at 590 nm (excitation 570 nm) using a Varioskan™ Multimode plate reader (Thermo Scientific).

## Results

### Novel imide substituted cycloheximide derivatives possess improved FKBP inhibitory activities

In the search for novel FKBP inhibitors without immunosuppressive side effects, we decided to extend the number of derivatives of the already described imide substituted cycloheximide compounds (Christner et al., 1999). Of the nine compounds generated for this study, two (MT_30.3 and MT_30.9) were produced using tert-butylamine as a substituting functionality, while the rest carried adamantane based substitutions (Table 1). The inhibitory activities of these compounds were evaluated in a protease coupled PPIase assay with the chromogenic peptide substrate Succinyl-Ala-Phe-Pro-Phe-4-nitroanilide using the human FKBP12 as well as Mip of *L. pneumophila*. Generally, all the substances inhibited FKBP12 more efficiently than Mip except for MT_30.9, which was with IC_50_-values of 36.1 ± 4.1 and 31.7 ± 7.4 μM for FKBP12 and Mip, respectively, also the least efficient inhibitor. Similarly, the most effective inhibitor of FKBP12, MT_30.32 (IC_50_ 0.18 ± 0.05 μM), had with 1.7 ± 0.2 μM the lowest IC_50_-value for Mip. This correlation between FKBP12 and Mip could also be observed for the second and third best inhibitors MT_30.51 and MT_30.79, respectively. However, in case of these derivatives, the IC_50_-values for Mip were about 25-fold higher than for FKBP12, hence, considerably higher than MT_30.32. The remaining compounds inhibited FKBP12 and Mip in the range of 1–2 and 1.5–34 μM, respectively (Table 1).

**Table 1:** **PPIase inhibitory activities of novel cycloheximide derivatives and their structures**.

Compound	Structure	IC_50_ (FKBP12)	IC_50_ (Mip)
MT_30.3	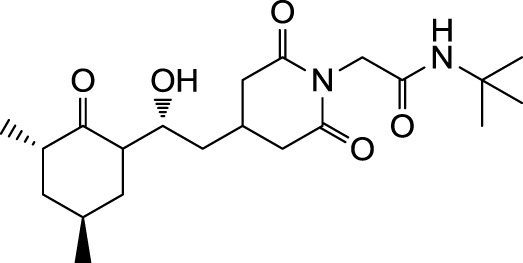	2.0 : 0.1 μ M	33.8 ± 8.6 μ M
MT_30.8	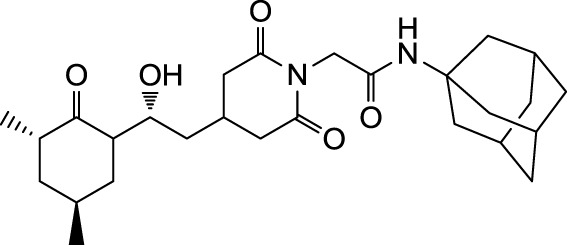	0.4 ± 0.04 μ M	8.5 ± 2.1 μ M
MT_30.9	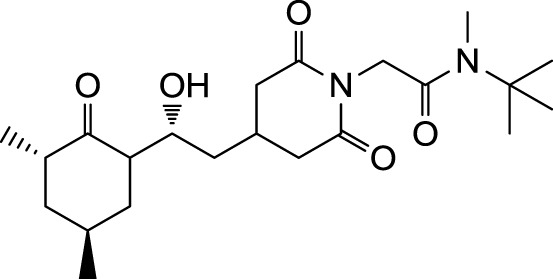	36.1 ± 4.1 μ M	31.7 ± 7.4 μ M
MT_30.32	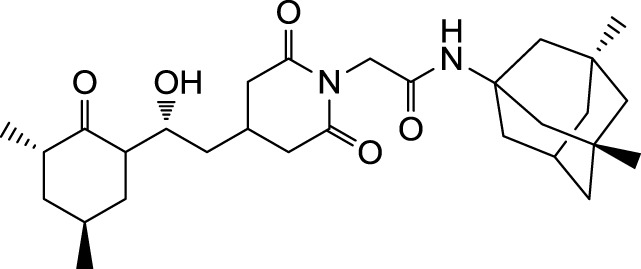	0.18 ± 0.05 μ M	1.7 ± 0.2 μ M
MT_30.38	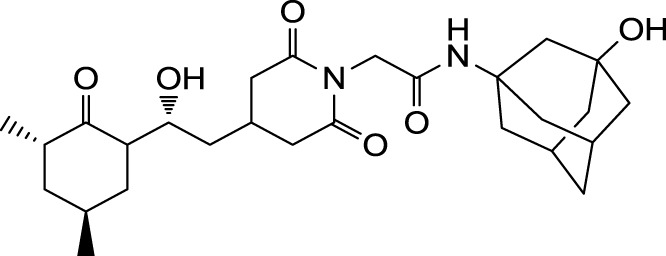	1.2 ± 0.1 μ M	21.8 ± 3.3 μ M
MT_30.51	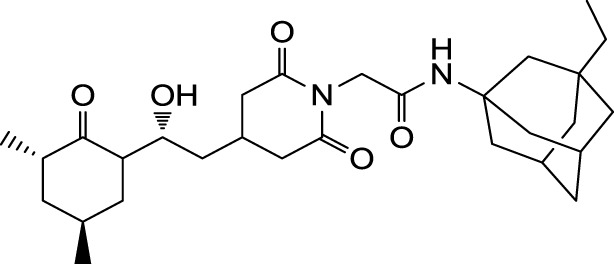	0.19 ± 0.06 μ M	5.6 ± 1.1 μ M
MT_30.79	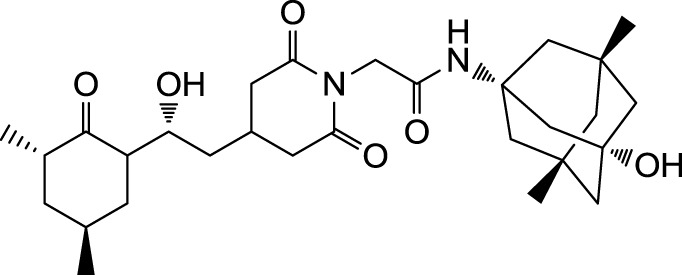	0.22 ± 0.02 μ M	4.9 ± 0.9 μ M
MT_30.92	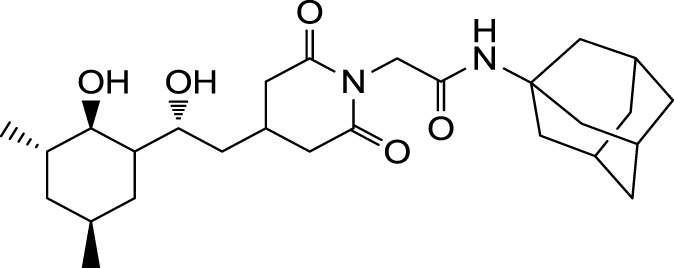	1.0 ± 0.1 μ M	12.6 ± 1.4 μ M
MT_30.93	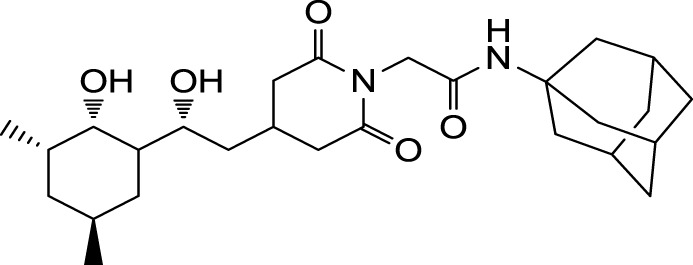	0.9 ± 0.05 μ M	16.3 ± 2.6 μ M

### Cycloheximide derivatives specifically inhibit growth of *L. pneumophila* in liquid culture

Since novel cycloheximide derivatives were able to inhibit the PPIase activity of Mip, we went on to evaluate the effect of these compounds on *Legionella* growth in liquid culture and during macrophage infection. For determining the MIC-values of the derivatives, wild type *L. pneumophila* and its isogenic Δ*mip*-mutant strain were cultured in the presence of the respective compound at final concentrations ranging from 10 to 100 μM. As a control, the known Mip inhibitor rapamycin was used. Substances MT_30.9, MT_30.32, and MT_30.51 inhibited the growth of *L. pneumophila* completely at a concentration of 30 μM, whereas, MT_30.92 and MT_30.93 started to inhibit at 40 μM. In contrast, the compounds MT_30.3, MT_30.8, MT_30.38, and MT_30.79 had no influence on the viability of wild type *L. pneumophila*. Remarkably, no differences could be observed in the spectrum and range of activity of the inhibitors between the *L. pneumophila* wild type and its isogenic *mip*-deficient mutant. Another interesting observation was that rapamycin also exhibited a growth inhibitory activity on both strains at a concentration of 40 μM or higher (Table 2).

**Table 2:** **MICs evaluated for *L. pneumophila* Corby and its isogenic *mip-*deficient mutant**.

Compound	WT		Δ*mip*	
	Inhibition^a^	MIC^b^	Inhibition	MIC
MT_30.3	-	-	-	-
MT_30.8	-	-	-	-
MT_30.9	+	30	+	30
MT_30.32	+	30	+	30
MT_30.38	-	-	-	-
MT_30.51	+	30	+	30
MT_30.79	-	-	-	-
MT_30.92	+	40	+	40
MT_30.93	+	40	+	40
Rapamycin	+	40	+	40

*^a^(-): no inhibition of growth at the highest tested concentration of 100 μM*.

*^b^Minimal inhibitory concentrations are given in μM*.

The strongest inhibitor in the PPIase assay, MT_30.32, was chosen as a representative, and further tested against the clinically relevant Gram-negative species *Y. pseudotuberculosis, P. aeruginosa* PA01*, S. enterica* subsp. *enterica, K. aerogenes*, and *E. coli* K12, as well as the Gram-positive pathogen *S. aureus*. To our surprise, none of the tested strains were affected in their growth by MT30_32 indicating that the antibacterial activity of our novel cycloheximide derivatives is either restricted to a narrow range of bacterial species or even specific against *L. pneumophila* (data not shown).

### Cycloheximide derivatives with adamantyl substitution inhibit *Legionella* replication during infection

**Figure 2:**
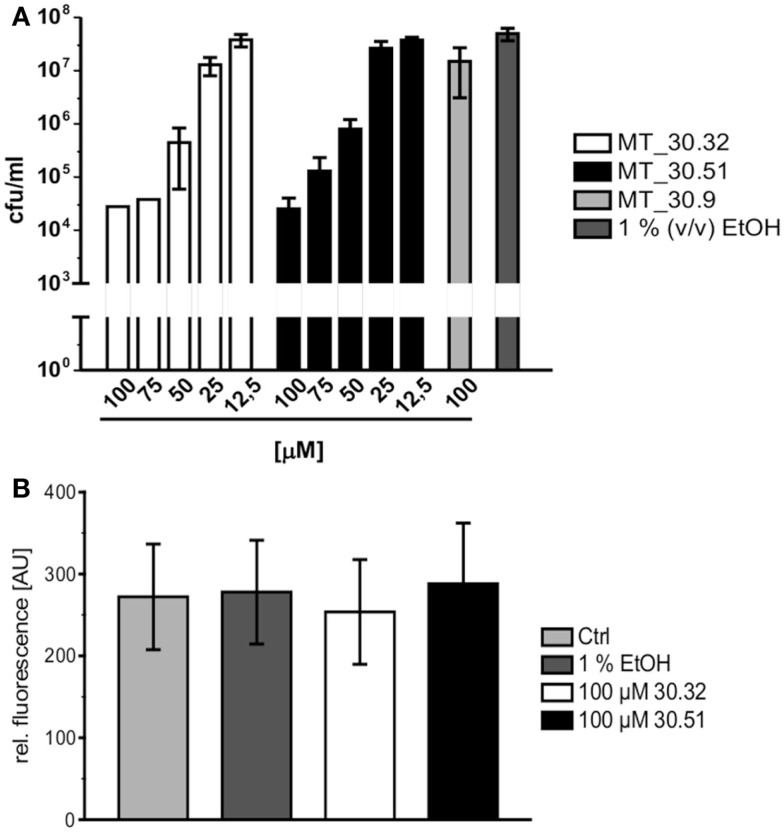
**Cycloheximide derivatives differentially inhibit bacterial replication during infection and are not cytotoxic**. **(A)** All inhibitors were tested in macrophage infection assays at concentrations ranging from 12.5 to 100 μM. The substances were added at the indicated concentrations 2 h post infection, and the bacterial replication was monitored by determining the colony forming units/milliliter (cfu/ml) 24 h post infection. The novel PPIase inhibitors MT_30.32 and MT_30.51 effectively suppressed bacterial replication during infection of differentiated THP-1 cells in a concentration dependent manner. The remaining seven derivatives had no effect at the highest concentration tested as demonstrated by the example MT_30.9, and were comparable to untreated infections containing only 1% (v/v) ethanol as the solvent at its final concentration. The graph depicts mean and SD of two independent experiments performed in duplicate as a representative of four biological replicates. **(B)** Differentiated THP-1 cells were incubated with 100 μM MT_30.32 or MT_30.51 for 24 h. After 20 h, alamar blue was added and cell viability was determined by measuring fluorescence at 590 nm. The medium of control cells was either free of additives (untreated) or contained 1% (v/v) EtOH as a solvent control. The graph shows the mean and SD of two independent experiments performed in triplicate.

During lung infection, *L. pneumophila* parasitizes human alveolar macrophages for its replication. Therefore, we tested whether the strongest inhibitors MT_30.32 and 30.51 were also capable of inhibiting bacterial replication during infection in differentiated THP-1 macrophages. In accordance with the protease coupled PPIase and MIC assays, both substances suppressed bacterial replication in THP-1 cells in a concentration dependent manner starting at 50 μM. In contrast, MT_30.9 that also inhibited bacterial growth but was about 18-fold less effective than MT_30.32 in the protease coupled PPIase-assay, had no effect on bacterial replication in THP-1 cells at the highest tested concentration of 100 μM (Figure 2A). None of the substances were cytotoxic to the THP-1 cells (Figure 2B).

## Discussion

The increase in antibiotic resistant strains and the spread of antibiotic resistance among disease causing organisms have put moonlighting proteins in the spotlight of the search for alternative drug targets, because their moonlighting properties are very often relevant for virulence (Henderson and Martin, 2014). The primary aim of this study was the design of non-macrolide, non-immunosuppressive inhibitors of the moonlighting virulence factor Mip of *L. pneumophila*. Mip originally was identified as a virulence factor that is needed during the early time points of intracellular replication in macrophages and protozoa (Cianciotto et al., 1989; Cianciotto and Fields, 1992; Fischer et al., 1992). While the PPIase activity affects invasion of monocellular cells, but not intracellular replication, a mutant strain expressing a Mip variant with very low PPIase activity is attenuated in the guinea pig infection model (Wintermeyer et al., 1995; Köhler et al., 2003). In accordance with observations in the animal model, Mip was shown to bind as a moonlighting protein collagen IV in the ECM of lung tissue. By doing this, it enables efficient colonization and dispersal of the bacteria to the spleen of infected animals extending its functions beyond the infection of monocellular hosts (Wagner et al., 2007).

Due to the high structural similarity between Mip and human FKBPs, like FKBP12, the design of Mip specific inhibitors benefits much from structural data and computational modeling. Until now two approaches for the design of Mip-specific inhibitors have been published. First, the moonlighting property was utilized, and the binding sequence within collagen IV was identified as being a 13-mer proline-rich peptide (P290) within the NC1-domain of collagen IV(α1). This peptide was chemically synthesized, and a computer model of the solution structure of the Mip-P290 complex could be generated following NMR measurements. Predictions based on this computer model allowed the design of a cyclic variant of P290 that was able to decrease bacterial transmigration across an *in vitro* epithelial cell barrier (Ünal et al., 2011).

In a second approach, Juli and coworkers synthesized novel pipecolic acid derivatives by starting from rapamycin, because computational analysis of the NMR structure of the Mip-rapamycin complex shows that rapamycin’s pipecoline moiety is anchored in the hydrophobic active site of Mip (Ceymann et al., 2008; Juli et al., 2011). The resulting compounds were found to inhibit the PPIase activity of Mip with IC_50_ values as low as 6 μM. However, the compounds inhibited the prototypic human FKBP12 30-fold more efficiently than Mip, indicating low specificity for bacterial FKBPs (Juli et al., 2011). In a follow-up study, the inhibitory activities of these derivatives were also confirmed for BpML1 and Mip-like proteins of the pathogens *Yersinia pestis* and *Francisella tularensis* (Begley et al., 2014).

For the design of novel inhibitors with increased specificity for Mip, we followed in the present study a new approach, and combined two previously described FKBP inhibitors by using synthetic chemistry. Of these inhibitors, cycloheximide was identified by classical functional screens, while adamantane was predicted by computer modeling, and its derivatives were validated experimentally (Babine et al., 1995; Christner et al., 1999). A general observation for the novel cycloheximide derivatives presented in this study was that imide substitution of cycloheximide with adamantyl derivatives yielded more efficient Mip inhibitors than substituting with *tert-*butylamine, since the highest IC_50_-value was 16.3 ± 2.6 μM for the former group (MT_30.93) compared to 31.7 ± 7.4 μM as the lowest IC_50_-value for the latter (MT_30.9) (Table 1).

Cycloheximide-*N*-ethylethanoate was reported to have an IC_50_-value of 6.5 ± 1 μM toward the Mip-like protein of *Burkholderia pseudomallei* (BpML1) (Norville et al., 2011). By using adamantyl derivatives as substituents, we were able to produce cycloheximide derivatives with improved IC_50_-values as low as 1.7 ± 0.2 μM in case of MT_30.32 supporting our combinatorial approach. Although the substances were, except for MT_30.9, more efficient inhibitors of FKBP12, the specificity toward Mip could also be increased compared to the previously described pipecolinic acid derivatives as MT_30.32 inhibited FKBP12 only 10-fold better.

**Figure 3:**
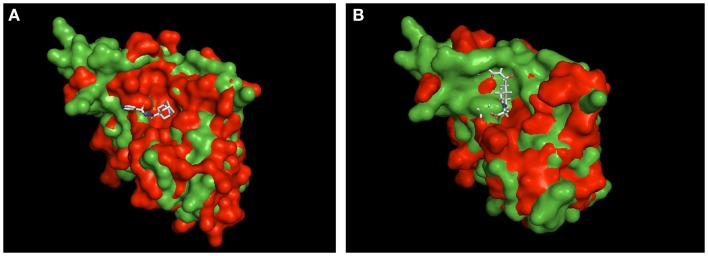
**Structural alignment of Mip to known PPIase-inhibitor complexes**. Shown are the structural overlays of the surface model of Mip (1FD9, green) with **(A)** the *Pv*FKBP35-SAR-complex (4MVG, red) and **(B)** the BpML1- cycloheximide *N*-ethylethanoate complex (2KO7, red). The ligands are shown as sticks. The overlay in **(A)** suggests that Mip and *Pv*FKBP35 are topologically more similar in the hydrophobic cleft, where SAR docks. Accordingly, a comparable binding mode of MT_30.32 or MT_30.51 to Mip via their adamantyl moiety can be assumed. The overlay in **(B)** reveals substantial topological differences between Mip and BpML1 in the loop region and the vicinity of the hydrophobic cleft that are reported to interact with cycloheximide N-ethylethanoate. Hence, the cycloheximide portions of MT_30.32 and MT_30.51 most probably bind in a different mode to Mip. The structural alignments were performed using PyMOL Molecular Graphics System, Version 1.5.0.4 Schrödinger, LLC.

Currently, we only can speculate about the binding mode of MT_30.32 and MT_30.51 to Mip. For cycloheximide-*N*-ethylethanoate, an NMR structure of its complex with BpML1 is published (2KO7). This revealed that the cycloheximide derivative displays a different binding mode than it is reported for FK506 and rapamycin. Rather than in the enzymatic cleft, cycloheximide-*N*-ethylethanoate interacts with residues in the close vicinity of it, and especially two loops outside the core (Norville et al., 2011). Similarly, a crystal structure of the adamantane derivative, supradamal, in complex with FKBP35 of *Plasmodium vivax* exists (4MGV). Here, the adamantane functionality perfectly fits into the hydrophobic cleft making non-bonded contacts with many conserved amino acids including Trp77 (Trp162 in Mip), which forms the base of the PPIase cavity (Harikishore et al., 2013). When these two structures are taken as reference and used for aligning Mip, it can be seen that the topological similarities between the PPIase domains of *Pv*FKBP35 and Mip are higher than BpML1 and Mip (Figure 3). Due to the higher similarity between *Pv*FKBP35 and Mip, it can be assumed that the adamantane moieties bind similarly to Mip by occupying the hydrophobic cavity (Figure 3A). In contrast, Mip and BpML1 differ especially in the region of the loop that is reported to interact with cycloheximide-*N*-ethylethanoate. Mip has a much bulkier topology at this part, which would affect the binding of cylocheximide inhibitors (Figure 3B). Accordingly, it would be very interesting for future studies to determine the binding mode of MT_30.32 or MT_30.51.

Having seen the improvement in Mip inhibitory capacity of the novel cycloheximide derivatives by achieving lower IC_50_-values and higher specificity, we evaluated their antibacterial potential in liquid growth and infection assays. Of the seven derivatives with adamantyl substitutions, four (MT_30.32, MT_30.51, MT_30.92, and MT_30.93) were able to inhibit bacterial growth in liquid culture starting at 30 μM (Table 2). Of those four, only two (MT_30.32 and MT_30.51) inhibited bacterial replication in macrophages without having cytotoxic side effects.

An interesting finding of our biological screens was that the inhibitory action of the four adamantyl substituted cycloheximide derivatives was independent of Mip, since a Δ*mip*-mutant was affected by the same compounds to the same extent. This indicates that there are additional drug target(s) than Mip. To our knowledge, there is no other FKBP in *L. pneumophila* except the ribosome associated trigger factor (TF) that contains a central FKBP domain (Rasch et al., 2014). However, the PPIase activity of TF is dispensable in many bacteria (Hoffmann et al., 2010). Hence, it is questionable, whether TF is the additional target of the biologically active derivatives. Apart from this, the facts that deletion of *mip* is not deleterious to the bacteria, but the inhibitors MT_30.32 and MT_30.51 are, nonetheless, effective during infection, suggest that some Mip functions might be taken over by other PPIases. A respective example has recently been described in *E. coli*, where deleting the periplasmic parvulin type PPIase SurA and the chaperone Skp is deleterious at 37°C due to the defects in the assembly of outer membrane proteins. However, under heat shock conditions this defect disappears because of the up-regulation of the periplasmic FKBP-type FkpA (Ge et al., 2014). Thus, it would be interesting for the future studies to test whether other *Legionella* PPIases can also be targeted by MT_30.32 and MT_30.51, or whether there might be alternative *Legionella* targets for these compounds. In conclusion, the adamantyl substituted cycloheximide derivatives presented in this study can be considered as a novel type of PPIase inhibitors with potential as antibacterial therapeutics.

## Conflict of Interest Statement

The authors declare that the research was conducted in the absence of any commercial or financial relationships that could be construed as a potential conflict of interest.
